# The audacious goal to end AIDS by 2030: aspiration or reality?

**DOI:** 10.1002/jia2.26339

**Published:** 2024-07-22

**Authors:** Quarraisha Abdool Karim, Kenneth H. Mayer, Jivanka Mohan, Carlos del Rio

**Affiliations:** ^1^ Centre for the AIDS Programme of Research, Nelson R Mandela School of Medicine University of KwaZulu‐Natal Durban South Africa; ^2^ Department of Epidemiology, Mailman School of Public Health Columbia University New York City New York USA; ^3^ The Fenway Institute of Health Boston Massachusetts USA; ^4^ Beth Israel Deaconess Medical Center Harvard Medical School Boston Massachusetts USA; ^5^ Division of Infectious Diseases and Center for AIDS Research Department of Medicine Emory University School of Medicine Atlanta Georgia USA

1

In 2015, world leaders pledged to end the AIDS epidemic as a public health threat by 2030 with the goals of zero new acquisitions, zero AIDS‐related deaths, and zero stigma and discrimination. It is undeniable that great strides have been made in initiating those living with HIV on antiretroviral medication (7.7 million in 2010 to 29.8 million in 2022) resulting in an estimated 51% reduction in AIDS‐related deaths (1.3 million in 2010 to 630,000 in 2022), a 58% decline in perinatal transmissions (310,000 in 2010 to 130,000 in 2022), and a 38% reduction in new acquisitions (2.1 million in 2010 to 1.3 million in 2022) [1]. However, the path ahead poses multiple challenges, particularly for ending new acquisitions and eliminating stigma and discrimination. The reality is that there is no room for complacency, as much remains to be done to get us to the UNAIDS 2030 goals. Furthermore, achieving these goals has become even more complicated because of disruptions in testing, treatment and support services caused by the COVID‐19 pandemic [2].

The global response to HIV/AIDS has been the most extraordinary and unprecedented public health endeavour in history. From the epidemic's bleakest days in the 1980s and early 90s, through the advent of life‐saving antiretroviral therapy in 1996, to global solidarity in the 2000s, we have seen the might of political will, science, activism and empathy [3].

Unfortunately, the phrase “ending the AIDS epidemic as a public health threat” has been heard by many—including political leaders, decision‐makers and the public—as “we have ended the AIDS epidemic,” which has resulted in unintended negative consequences, including threats to end programmes such as the Global Fund to Fight AIDS, Tuberculosis, and Malaria [4] and the President's Emergency Plan for AIDS Relief (PEPFAR) [5]. Achieving the 2030 goals will require renewed political commitment, innovative approaches and collaboration across sectors and borders—an all‐of‐society approach. In the absence of a vaccine and a cure, funding will be necessary beyond 2030 to sustain the gains, maintain 30 million people on treatment, support medication adherence, strengthen primary prevention including pre‐exposure prophylaxis (PrEP) and continue research to find effective vaccines and cures. Communicating this message to politicians, decision‐makers, funders and the public is a priority imperative.

Significant governmental and foundation investments have been made in research to find a vaccine and a cure for HIV [6], but more work needs to be done. Additionally, the development of new antiretroviral drugs and long‐acting formulations is necessary to improve adherence and clinical outcomes [7] as well as to increase uptake and persistent use among PrEP users. But the incentives for the industry to continue to innovate are predicated on the assumption that there will be resources available to support access to new medications.

One of the key achievements in four decades of the AIDS response is universal access to treatment [8], with the dual benefits of individual survival and prevention of onwards transmission of HIV. The social and economic impact of treatment as prevention has been substantial, but primary prevention remains a challenge. The potential for prevention with PrEP is yet to be realized, particularly in settings or populations where the 95‐95‐95 targets have been reached but rates of new acquisitions remain high [9]. The recently released findings from the PURPOSE‐1 trial [10] of complete protection for women with a 6‐monthly injection of a capsid inhibitor, lenacapavir, could be a real game changer for women and others globally—and particularly for young women in sub‐Saharan Africa who bear the brunt of the burden of new acquisitions, but who have limited power to negotiate safer sex practices with their male partners. However, to achieve the optimal benefits of PrEP, innovative systems will need to be put in place to deliver it in an affordable and accessible manner. While innovation remains central, we must focus on scaling up proven interventions.

The impact of programmes to prevent vertical transmission of HIV has been another major success [11, 12]. The challenge now is reaching pregnant women who do not use prenatal health services and arrive at facilities at the time of delivery with an unknown HIV status [13]. As with antiretroviral treatment, success to date has been dependent on clients utilizing health facilities. Attention also must turn to those who we are missing. This will require novel approaches and partnerships with the community.

Community involvement is key to the effectiveness of HIV programmes. Empowering local communities and involving them in planning and implementation ensures that interventions are culturally appropriate, inclusive and effective [13]. Community‐led initiatives address specific local challenges and improve the reach and impact of HIV services, for example through peer adherence support groups and community collection points for multi‐month dispensing of medication.

Efforts to reduce stigma and discrimination are essential for ensuring that people living with and at risk of HIV can access the care and support they need, including programmes to protect human rights [14]. Some countries have reformed laws and policies to protect the rights of people living with HIV, sex workers, people who use drugs and LGBTQ+ people, encouraging them to seek testing and treatment without fear of discrimination, while others have reversed progressive legislation. Since 2023, there has been a surge in discriminatory legislation directed against LGBTQ+ persons across Africa. Today, in 33 out of 55 African countries, homosexuality is a crime punishable by imprisonment, and in some countries like Uganda, the law includes the death penalty [15]. Sex work and drug use also remain criminalized activities. Reversing regressive legislation and/or decriminalization is critical to meeting the 2030 goals.

Focusing on getting all countries on the trajectory to meeting the 2030 goals is even more important now. To achieve them, we should focus on intensifying efforts by scaling up proven interventions and reaching populations most at risk. Sustained commitments from both international donors and domestic governments are crucial for ensuring long‐term gains are maintained [16]. Addressing social determinants of health and reducing disparities in access to healthcare services will help reduce inequities. Enhancing collaboration between governments, civil society, the private sector and international organizations is essential for strengthening partnerships [17]. By building on current successes, addressing persistent gaps, and maintaining a comprehensive and inclusive approach, the global community can move closer to achieving the 2030 goals.

Without a doubt, the path ahead remains steep, particularly for ending new acquisitions and eliminating stigma and discrimination for key and vulnerable populations. However, we hope that with unwavering effort, political commitment and global solidarity, the goals of zero new acquisitions, zero AIDS‐related deaths, and zero stigma and discrimination within this decade could still be achievable.

## COMPETING INTERESTS

The authors declare no potential conflict of interest with respect to the research, authorship and/or publication of this article. Disclosures: QAK, KM and CdR are members of the PEPFAR Scientific Advisory Board.

## AUTHORS’ CONTRIBUTIONS

QAK, KHM, JM and CdR contributed equally to drafting and finalizing this article.
